# A chromosome-level genome assembly of the *Knoxia roxburghii* (Rubiaceae)

**DOI:** 10.1038/s41597-023-02725-8

**Published:** 2023-11-15

**Authors:** Yingmin Zhang, Fan Zhang, Ling Jin, Ticao Zhang, Xinying Pu, Bin Qiu, Guodong Li

**Affiliations:** College of Chinese Material Medica, Yunnan University of Chinese Medicine, Kunming, 650500 China

**Keywords:** Plant genetics, Genomics, Comparative genomics

## Abstract

*Knoxia roxburghii* is a well-known medicinal plant that is widely distributed in southern China and Southeast Asia. Its dried roots, known as hongdaji in traditional Chinese medicine, are used to treat a range of diseases, including cancers, carbuncles, and ascites. In this study, we report a de novo chromosome-level genome sequence for this diploid plant, which has a length of approximately 446.30 Mb with a contig N50 size of 42.26 Mb and scaffold N50 size of 44.38 Mb. Approximately 99.78% of the assembled sequences were anchored to 10 pseudochromosomes and 3 gapless assembled chromosomes were included in this assembly. A total of 24,507 genes were annotated, along with 68.92% of repetitive elements. Overall, our results will facilitate further active component biosynthesis for *K. roxburghii* and provide insights for future functional genomic studies and DNA-informed breeding.

## Background & Summary

*Knoxia roxburghii* (Sprengel) M. A. Rau (2n = 20, homotypic synonym: *Knoxia valerianoides* Thorel ex Pitard), a perennial herb naturally distributed in southern China and Southeast Asia, is a member of the Rubiaceae family and the *Knoxia* genus^1^. The dried roots of *K. roxburghii*, known as hongdaji in Chinese medicine, exhibit a significant therapeutic effect in treating cancer, carbuncles, diarrhoea, ascites, chronic pharyngitis, and schizophrenia^2^. Additionally, the plant is a crucial ingredient in various Chinese herbal formulations, such as ZiJinDing, which has been shown to possess antitumour properties by modern pharmacology^3^. Phytochemical studies have revealed that *K. roxburghii* is rich in anthraquinones, triterpenoids, lignans, coumarins, sitosterols, and other important compounds^4,5^. Anthraquinones, such as 3-hydroxymoridone, knoxiadin, and damnacanthal, are considered key active components of *K. roxburghii*, exhibiting diverse biological activities including anticancer, antibacterial, anticoagulant, and antiviral effects^6,7^. Triterpenoids, which are a significant component of *K. roxburghii*, have anti-inflammatory, anticancer, and antioxidant effects. They are primarily responsible for reducing inflammation and swelling in *K. roxburghii*^8,9^.

In recent years, the wild populations of *K. roxburghii* in China have been facing an increased risk of extinction due to a surge in market demand^10^. Additionally, seed germination and emergence rates for this species are less than 1% under natural conditions, and it exhibits a protracted maturation period^11^. *K. roxburghii* has been categorized as a first-class protected wild Chinese herbal medicine, and its production area has been prohibited from being utilized^12^. As a result, artificially cultivated *K. roxburghii* has become the primary source of medicinal materials. Nevertheless, the cultivation process is plagued by southern blight and leaf spot, which have severely limited the plant’s production^13^. Therefore, there is an urgent need for the breeding of promising new *K. roxburghii* varieties to tackle this issue.

Whole‐genome-level studies can provide insights for enhancing medicinal material quality, molecular breeding, wild resource conservation, and functional gene discovery and utilization of plants^14–16^. However, to date, no whole-genome sequence of *K. roxburghii* has been reported. In the present study, by using DNBSEQ sequencing, single-molecule real-time sequencing, and high-throughput chromosome conformation capture sequencing (Hi-C) sequencing technologies, we provide a *de novo* high-quality chromosome-level genome sequence for *K. roxburghii*. The 99.78% genome sequence is anchored to 10 chromosomes, with a total length of 446.30 Mb and scaffold N50 of 44.38 Mb. Transposable elements accounted for 68.92% (307.60 Mb) of the assembled genome sequence, with long terminal repeats (LTRs) being the dominant type. The LTR retrotransposon burst was estimated to have occurred approximately 0.2 million years ago. Phylogenetic analysis revealed that Copia and Gypsy elements could be grouped into eight and five lineages, respectively. The reference genome information obtained herein constitutes a valuable resource for promoting genetic improvement and elucidating the biosynthesis of active ingredients in this medicinal plant.

## Methods

### Sample collection and sequencing

For genomic DNA extraction, fresh leaves of *K. roxburghii* were collected from Chuxiong (N24°58′, E101°28′) in Yunnan Province, China. Additionally, stems, roots, buds, and leaves were gathered to perform transcriptome sequencing. The materials were immediately preserved in liquid nitrogen, transported to the laboratory, and stored at −80 °C. High-quality genomic DNA was extracted from leaves using the DNeasy Plant Mini Kit (QIAGEN, Valencia, California, USA). Total RNA was extracted from each sample using the Directzol RNA kit (Zymo Research, Irvine, CA, USA) following the manufacturer’s instructions.

For short-reads sequencing, paired‐end DNBSEQ libraries were constructed using the NextEra DNA Flex Library Prep Kit (Illumina, San Diego, CA, USA) with an insert size of 350 bp and sequenced on the DNBSEQ-T7 platform (MGI Tech, Shenzhen, China). A quality assessment of the short sequencing reads was conducted using fastp v. 0.21.0^17^ with default parameters. This process involved the removal of adapter sequences, contaminants, PCR duplicates, and reads with a low-quality base percentage exceeding 30%. A total of 107.86 Gb clean short reads (251.78 × coverage) were generated and used for subsequent data processing. The genome size was estimated to be 428.39 Mb, with a heterozygosity of 1.23% and repetitive content of 46.86% based on previous K-mer distribution analyses^18^.

For PacBio sequencing, the libraries were constructed with an insert size of 15 kb using the SMRTbell Template Prep Kits (Pacific Biosciences of California, Inc., CA, USA) and sequenced in CCS mode on the PacBio Sequel II platform (continuous long reads (CLR) sequencing mode). After trimming the low-quality reads and adaptor sequences from the raw data, approximately 52.85 Gb of long reads were generated, covering approximately 124 × of the estimated genome size.

For Hi-C sequencing, the library was prepared according to the protocol described by Lieberman-Aiden^19^ *et al*. DNA was purified from proteins and randomly sheared into fragments of 300–700 bp in size. The resulting Hi-C library was sequenced on the Illumina NovaSeq 6000 sequencing platform using paired-end 150 bp reads. The raw data from Hi-C sequencing were processed using fastp. A total of 36.14 Gb (84.36 × coverage) of clean reads were obtained.

For Oxford Nanopore Technologies (ONT) sequencing, all RNA samples of the same quantity were mixed for PCR-cDNA library construction using the Ligation Sequencing Kit (SQK-LSK109) and sequenced on the PromethION sequencer (Oxford Nanopore Technologies, Oxford, UK). NanoFilt v. 2.8.0^20^ (parameters: –q 7 –l 100 –headcrop 30 –minGC 0.3) was used to process the RNA-seq data. Finally, a total of 6.2 Gb of full-length RNA-seq data were obtained for genome annotation.

### Genome and chromosome assembly

The contig-level genome of *K. roxburghii* was assembled using Hifiasm v. 0.14.2^21^ with default parameters. Two rounds of error correction were performed based on PacBio sequencing and Illumina NovaSeq sequencing data using NextPolish v. 1.3.1^22^ (parameters: sgs_options = –max_depth 200 lgs_options = –min_read_len 1k –max_read_len 100k –max_depth 100 lgs_minimap2_options = –x map-ont) and Pilon v. 1.23^23^ (parameters:–fix all–changes), respectively. The heterozygous sequences were removed by using the Purge_haplotigs pipeline v. 1.0.4^24^. The high, mid, and low cut-off read depth parameters were set to 170, 55, and 5, respectively, to remove haplotigs. Consequently, the genome assembly contained 446.30 Mb in 19 contigs with a contig N50 of 42.26 Mb, and the GC content of the genome was 35.98% (Table 1).

**Table 1:** Global statistics for the *Knoxia roxburghii* genome assembly.

Genome features	Size/Number	
	Contig	Scaffold
Total number	19	16
Total length (bp)	446,302,636	446,303,736
Max (bp)	45,435,640	48,324,875
Average (bp)	23,489,612.42	27,893,983.50
Min (bp)	56,849	5,000
N50 (bp)	42,264,240	44,377,471
N90 (bp)	34,037,809	42,264,240

**Fig. 1:**
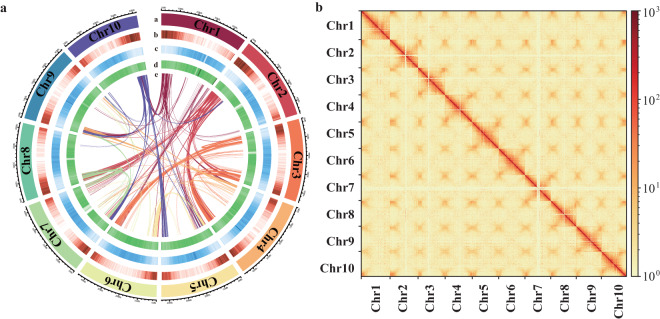
Overview of the genomic features of *Knoxia roxburghii*. (**a**) Genomic features of *K. roxburghii*. Tracks from outside to inside (**a**–**e**) are as follows: chromosomes, gene density, repeat sequence density, GC content, and collinearity between the chromosomes; (**b**) Hi-C interaction heatmap for the *K. roxburghii* genome showing interactions among the ten chromosomes.

The Hi-C clean data were mapped to the draft genome using HiCUP v. 0.8.2^25^ (parameters: –format sanger –longest 800 –shortest 150 –nofill N), followed by filtration to remove unmapped reads, invalid pairs, and PCR amplification-induced repetitive sequences. ALLHiC v. 0.9.8^26^ (parameters: –e GATC –k 10) was utilized to cluster the contigs into chromosomal groups, with subsequent sorting and orientation. The interactions between contigs were converted into a specific binary file using 3D-DNA v. 180419^27^ and Juicer v. 1.6^28^. Then, the visual correction of the assembly was finalized using JuiceBox v. 1.11.08^29^ based on the intensity of chromosome interaction. Additionally, very short contigs without any interaction relationships were placed in the “unassigned” category. The final chromosomal-level genomic sequence was obtained by using 100 N to fill the gaps. Finally, 99.78% of the initial assembled sequences were anchored to 10 pseudo-chromosomes with lengths ranging from 42.02 Mb to 48.32 Mb (Fig. 1a, Table 2). The total length of the genome assembly was 446.30 Mb, with a scaffold N50 of 44.38 Mb (Table 1).

**Table 2:** Statistics of the pseudochromosome length obtained by Hi-C assisted assembly of *Knoxia roxburghii*.

Pseudochromosome	Length (bp)	Contig number
Chr1	48,324,875	3
Chr2	47,144,680	3
Chr3	45,444,268	3
Chr4	45,435,640	1
Chr5	44,377,471	2
Chr6	44,179,751	3
Chr7	43,311,066	1
Chr8	42,833,556	2
Chr9	42,264,240	1
Chr10	42,023,723	2

### Genome annotations

Three gene prediction methods, namely *de novo*-based, RNA-seq-based, and homologue-based, were combined to identify gene structures. For *de novo*‐based prediction, gene prediction was performed using AUGUSTUS v. 3.2.3^30^ and GlimmerHMM v. 3.0.4^31^ with default parameters. In the RNA-seq-based approach, the full-length sequence underwent alignment to the reference genome using Minimap2 v. 2.17^32^ (parameters: –ax map-ont –xsplice –G 1000000). Subsequently, the alignment results were used as inputs in StringTie v. 1.3.3^33^ for genome-based transcript assembly, and coding regions were then predicted using TransDecoder v. 2.0 (http://transdecoder.github.io). Homology‐based predictions were performed with protein sequences from five reference species: *Arabidopsis thaliana*^34^, *Coffea arabica*^35^, *Coffea canephora*^36^, *Leptodermis oblonga*^37^, and *Mitragyna speciosa*^38^. The results of the three methods were integrated using MAKER v. 2.31.10^39^. Overall, a total of 24,507 genes have been successfully predicted, with an average gene length, average coding-sequence length, average exon length, and average exon number per gene of 4036.6 bp, 1205.64 bp, 318.24 bp, and 5.14, respectively (Table 3).

**Table 3:** Statistical results for the genetic structure of *Knoxia roxburghii*.

Item	Number
Total number of genes	24,507
Average of mRNA length (bp)	4,036.60
Average CDS length of per gene (bp)	1,205.64
Average exon number of per gene	5.14
Average of exon length (bp)	318.24
Average of intron length (bp)	574.92
Total number of exons	125,865
Total number of introns	101,358
Total intron length (bp)	58,273,234

Gene functions were assigned to the protein-coding gene models and compared to the National Center for Biotechnology Information (NCBI) Non-redundant protein (NR) (ftp://ftp.ncbi.nih.gov/pub/nrdb/), the Universal Protein Knowledgebase (UniProt) database^40^, and the Kyoto Encyclopedia of Genes and Genomes (KEGG) database^41^ using diamond v. 2.0.11.149^42^ (parameters: –evalue 1e-5). The motifs and domains were identified using InterProScan v. 5.52-86.0^43^ against multiple publicly available databases including ProDom^44^, PRINTS^45^, Pfam^46^, SMRT^47^, PANTHER^48^, and PROSITE^49^. A total of 24,236 genes (94.85% of the predicted protein-coding genes) were annotated using the above databases. Specifically, approximately 90.88%, 91.06%, 25.34%, 92.88%, 70.87%, and 69.22% were annotated in UniProt, Nr, KEGG, InterPro, GO, and Pfam, respectively.

The identification of transfer RNAs (tRNAs) was performed using tRNAscan-SE v. 2.0.7^50^. Other non-coding RNAs (ncRNAs), such as microRNAs (miRNAs) and small nuclear RNAs (snRNAs), were identified using Infernal v. 1.1.2^51^ by searching against the Rfam database^52^. Lastly, the number of rRNAs, snRNAs, miRNAs, and tRNAs predicted from *K. roxburghii* genome were 1,053, 550, 81, and 387, respectively (Table 4).

**Table 4:** Statistics of non-coding RNA prediction in the *Knoxia roxburghii* genome.

	Types	Number	Average length (bp)	Total length (bp)	Percentage (%)
miRNA		81	133	10,778	0.002415
tRNA		387	75	29,162	0.006534
rRNA	rRNA	1,053	155	162,924	0.036505
	18 S	21	1,759	36,941	0.008277
	28 S	43	199	8,559	0.001918
	5.8 S	23	155	3,572	0.0008
	5 S	966	118	113,852	0.02551
snRNA	snRNA	550	108	59,434	0.013317
	CD-box	286	101	28,757	0.006443
	HACA-box	42	128	5,376	0.001205
	splicing	222	114	25,301	0.005669

### Transposable elements and annotation of repeat sequences

Repetitive elements were identified through transposable element annotation using the Extensive *de novo* TE Annotator (EDTA) program v. 2.0.1^53^ (parameters:–sensitive 1–anno 1). The insertion time was calculated using the LTR_retriever^54^ with default parameters. TEsorter v. 1.3^55^ (parameters: -db rexdb) was used to classify the clade level of LTR-RTs and extract LTR-RT protein domains. MAFFT v. 7.475^56^ (parameters:–auto) was utilized to align LTR-RT sequences, and a phylogenetic tree was constructed using IQ‐TREE v. 2.2.2.6^57^ (parameters: –bb 1000).

**Fig. 2:**
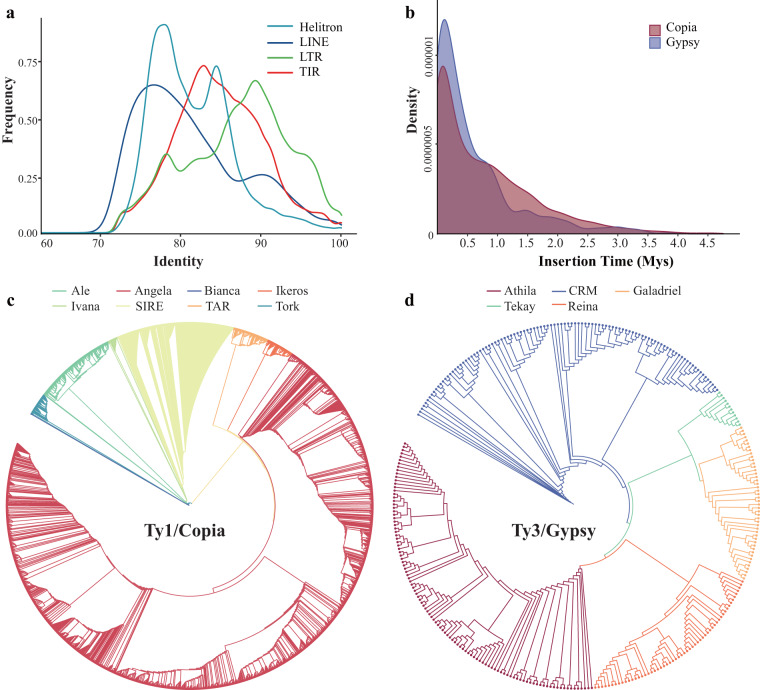
Repeat sequence analysis of the *Knoxia roxburghii* genome. (**a**) Distribution of sequence identity values between genomic copies and consensus repeats in the *K. roxburghii* genome. (**b**) Distribution of sequence identity values between genomic copies and consensus repeats in the *K. roxburghii* genome. (**c**) Phylogenetic tree of Ty1/Copia-type retrotransposons. (**d**) Phylogenetic tree of Ty3/Gypsy-type LTR retrotransposons.

Based on the high-quality reference genome in this study, 307.60 Mb of repetitive sequences of *K. roxburghii* were predicted (Table 6). Among the integrated results, 33.56% (149.76 Mb) of the sequences were long terminal repeat (LTR) retrotransposons, with LTR/Copia elements being the dominant class (28.71% of the whole genome, 128.15 Mb), followed by LTR/Gypsy elements (2.79% of the whole genome, 12.47 Mb). To investigate the evolutionary history of transposable elements (TEs) in the *K. roxburghii* genome, a distribution plot of identity values between genomic copies and their consensus sequences was generated. The distributions of LTRs showed a peak at 89% identity, which was larger than the peaks of the other TE types, indicating that LTR-retrotransposons were recently transposed in the genome of *K. roxburghii* (Fig. 2a). Additionally, the genome contained 3,394 LTR-RTs, and the LTR retrotransposon burst was estimated to have occurred approximately 0.2 million years ago (Fig. 2b). For LTR/Gypsy and LTR/Copia, phylogenetic trees revealed that repeat elements were organized into different clades and expanded in clusters (Fig. 2c,d).

## Data Records

The BGI short reads, PacBio HiFi long-reads, Hi-C reads, and RNA-Seq data have been deposited in the NCBI Sequence Read Archive with accession numbers SRR25777372^58^, SRR25787934^59^, SRR24958413^60^, and SRR25775167^61^. The genome assembly has been deposited in DDBJ/ENA/GenBank under the accession number JAUECX000000000^62^. The chromosomal assembly and dataset of gene annotation have been deposited in the FigShare database at 10.6084/m9.figshare.23542566^63^.

## Technical Validation

The integrity of the genome assembly was assessed using the sequence identity method. Reads from a small-fragment library were specifically selected and aligned to the assembled genome using BWA v. 0.7.17-r1188^64^. The alignment rate of all small fragment reads to the genome was approximately 99.60%, and the coverage rate was approximately 99.49%, indicating consistency between the reads and the assembled genome.

We performed a Benchmarking Universal Single-Copy Orthology (BUSCO) v. 4.1.4^65^ analysis based on the embryophyta_odb10 database to assess the completeness of the assembly, which indicated that 97.50% of the complete BUSCOs were present in the assembly (Table 5). Furthermore, 99.78% of the scaffolds were successfully anchored to the 10 chromosomes. The accuracy of the chromosome assembly was indirectly confirmed by examining the Hi-C heatmap, which revealed a well-organized interaction contact pattern along the diagonals within and around the chromosome region (Fig. 1b). This observation provides additional support for the precision of the chromosome assembly.

**Table 5:** Statistics for BUSCO estimation for *Knoxia roxburghii* genome assembly and annotation.

Types	Assembly		Annotation	
	Number	Percentage (%)	Number	Percentage (%)
Complete BUSCOs (C)	1,575	97.50	1,588	98.40
Complete and single-copy BUSCOs (S)	1,534	95.00	1,549	96.00
Complete and duplicated BUSCOs (D)	41	2.75	39	2.40
Fragmented BUSCOs (F)	10	0.46	7	0.40
Missing BUSCOs (M)	29	1.90	19	1.20
Total BUSCO groups searched	1,614	100.00	1,614	100.00

**Table 6:** Statistics of repeat elements of the *Knoxia roxburghii* assembly.

Type	TE proteins		*De novo* + Repbase		Combined TEs	
	Length (bp)	Percentage (%)	Length (bp)	Percentage (%)	Length (bp)	Percentage (%)
DNA	2,939,793	0.66	32,345,797	7.25	32,723,525	7.33
LINE	1,743,846	0.39	6,279,415	1.41	6,539,492	1.47
SINE	0	0	8,853	0	8,853	0
LTR	68,552,820	15.36	146,163,305	32.75	149,761,257	33.56
LTR-Gypsy	5,147,193	1.15	12,178,188	2.73	12,468,090	2.79
LTR-Copia	58,375,581	13.08	127,323,355	28.53	128,154,235	28.71
Satellite	0	0	62,933	0.01	62,933	0.01
Simple repeat	0	0	115,868	0.03	115,868	0.03
Other	396	0	2,130	0	2,526	0
Unknown	14,928	0	127,099,848	28.48	127,114,776	28.48
Total	73,217,809	16.41	300,115,040	67.24	307,601,852	68.92

To validate the predicted genes, we performed a BUSCO analysis. The analysis revealed a high reliability of the annotated results, as approximately 98.40% of the complete BUSCOs were identified (Table 5). The annotation results were considered acceptable since the number of predicted genes and structural characteristics of the *K. roxburghii* genome were consistent with those of the genomes of closely related species.

## Data Availability

All software and pipelines were executed according to the manual and protocols of the published bioinformatics tools. The version and code/parameters of the software have been detailed and described in Methods. No custom code was used during the compilation of the dataset.
